# Using Interleaved Stimulation and EEG to Measure Temporal Smoothing and Growth of the Sustained Neural Response to Cochlear-Implant Stimulation

**DOI:** 10.1007/s10162-023-00886-2

**Published:** 2023-02-08

**Authors:** François Guérit, John M. Deeks, Dorothée Arzounian, Robin Gransier, Jan Wouters, Robert P. Carlyon

**Affiliations:** 1 Cambridge Hearing Group, MRC Cognition & Brain Sciences Unit, University of Cambridge, Cambridge, England; 2 ExpORL, Dept. of Neurosciences, KU Leuven Leuven, Belgium

**Keywords:** Cochlear implant, EEG, Electrophysiology, ALFIES, Neural distortion response, EASSR

## Abstract

Two EEG experiments measured the sustained neural response to amplitude-modulated (AM) high-rate pulse trains presented to a single cochlear-implant (CI) electrode. Stimuli consisted of two interleaved pulse trains with AM rates F1 and F2 close to 80 and 120 Hz respectively, and where F2 = 1.5F1. Following Carlyon et al. (*J Assoc Res Otolaryngol*, 2021), we assume that such stimuli can produce a neural distortion response (NDR) at F0 = F2-F1 Hz if temporal dependencies (“smoothing”) in the auditory system are followed by one or more neural nonlinearities. In experiment 1, the rate of each pulse train was 480 pps and the gap between pulses in the F1 and F2 pulse trains ranged from 0 to 984 µs. The NDR had a roughly constant amplitude for gaps between 0 and about 200–400 µs, and decreased for longer gaps. We argue that this result is consistent with a temporal dependency, such as facilitation, operating at the level of the auditory nerve and/or with co-incidence detection by cochlear-nucleus neurons. Experiment 2 first measured the NDR for stimuli at each listener’s most comfortable level (“MCL”) and for F0 = 37, 40, and 43 Hz. This revealed a group delay of about 42 ms, consistent with a thalamic/cortical source. We then showed that the NDR grew steeply with stimulus amplitude and, for most listeners, decreased by more than 12 dB between MCL and 75% of the listener’s dynamic range. We argue that the NDR is a potentially useful objective estimate of MCL.

## Introduction

Noninvasive measures of the neural response to electrical stimulation by a cochlear implant (CI) may bring clinical and scientific benefits. Clinically, these objective measures may inform the fitting of CIs in patients, such as young children, who cannot make reliable behavioural responses, and potentially help the clinician understand why a particular patient gains little benefit from their device. Scientifically, a noninvasive neural measure could be applied to studies of novel interventions, such as optogenetic or intra-neural stimulation, that are initially applied only to nonhuman species [1, 2]. In both cases, it is desirable to use stimuli that are as close as possible to those applied by patients’ CIs to convey speech and other sounds in everyday use. Unfortunately, the large electrical artefact produced by CI stimulation has required researchers and clinicians to compromise on this last requirement. For example, the two most widely adopted clinical measures are the electrically evoked compound action potential (ECAP) and auditory brainstem responses (EABRs), both of which present single pulses at a slow rate (< 100 pps) and measure the neural responses in the gaps between the pulses. These stimuli differ markedly from the high-rate amplitude-modulated (AM) pulse trains that CIs employ to convey speech sounds, and the correlation between loudness levels with high-rate AM stimuli and both ECAPs and EABRs is quite low [3–7].

**Fig. 1:**
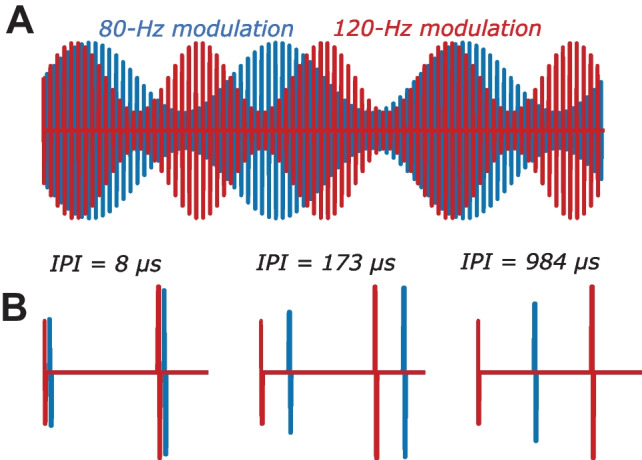
**A **Schematic of the stimuli used in experiment 1 and 2, showing interleaved pulse trains amplitude-modulated at F1 = 80 Hz and F2 = 120 Hz. Part **B** shows zoomed-in portions illustrating the effects of inter-pulse interval (IPI) as measured in experiment 1

We recently described a new way of measuring the sustained neural response to high-rate AM pulse trains applied to one or more CI electrodes. The Alternating Low-Frequency Interleaved Stimulation (ALFIES) method [8] interleaves the pulses from two sinusoidally amplitude-modulated pulse trains, having identical pulse rates and different modulation frequencies of F1 and F2 Hz (Fig. 1A). When F1 and F2 were about 80 and 120 respectively, EEG recordings revealed the presence of a frequency component at about 40 Hz; this was equal to both F2-F1 Hz and to 2F1-F2 Hz, and so could have reflected either a quadratic and/or cubic distortion component. We termed this component the neural distortion response (NDR) and concluded that it arose from nonlinearities inherent to the auditory system’s response to electrical stimulation. We argued that it did not reflect nonlinearities in the CI stimulus, because those nonlinearities should operate almost instantaneously and because pulses from the F1-Hz and F2-Hz AM pulse trains were interleaved in time. The independence produced by interleaving the two pulse trains may be undone by numerous known temporal dependencies in the neural response, including charge integration and facilitation at the auditory-nerve membrane and phenomena such as co-incidence detection, refractoriness, and adaptation that occur in the auditory system. We reasoned that once the interleaving has been undone by such temporal dependencies, an NDR can then be generated by nonlinearities at any subsequent stage of auditory processing. The neural basis of the NDR was demonstrated by its group delay of about 45 ms, consistent with a thalamic and/or cortical source, its similar size for recording electrodes close to or far from the CI, and the fact that it was not observed when F1 and F2 were increased to about 220 and 330 Hz respectively. This pattern differed markedly from the artefact response at F1 and F2 Hz, which had zero latency, an amplitude that differed by about 20 dB across recording electrodes, and that was of roughly equal amplitude for all values of F1 and F2 tested. We also showed that presenting the F1 and F2 pulse trains to different electrodes produced an NDR whose amplitude decreased with increasing electrode separation, thereby providing a measure of the selectivity of electrically evoked neural excitation.

Here, we present two validations and extensions of the ALFIES method. Experiment 1 measures the NDR as a function of the temporal gap between the pulses in the F1 and F2 pulse trains (Fig. 1B). We report that the NDR is roughly constant for inter-pulse intervals (IPIs) shorter than about 200–400 µs and gradually decreases as IPI is increased further, being unmeasurable at IPI > 900 µs. We argue that this time course is consistent with temporal smoothing occurring as early as charge integration at the auditory-nerve membrane [9–14], and might additionally reflect co-incidence detection of auditory-nerve action potentials in the cochlear nucleus [15–18]. Experiment 2 measures the effect of stimulus level on the amplitude of the NDR. We report that the amplitude growth functions are steep and that an NDR is usually observed only when the level falls within the top 25% of the listener’s dynamic range. We argue therefore that the ALFIES method may provide a useful objective estimate of a listener’s most comfortable level (MCL) for a given electrode. Both experiments extend our previous use of ALFIES with listeners of the CI manufactured by the Cochlear company to those implanted with an Advanced Bionics CI, and implement a real-time measure of signal-to-noise ratio (SNR) that can reduce recording time to between 3 and 4 min.

## Methods

### General

Both experiments involved direct stimulation of a single CI electrode using specialised research software and hardware provided by the manufacturers. A total of 11 participants, all implanted with either Cochlear or Advanced Bionics CIs, took part. Their details are provided in Table 1. Experimental procedures were approved by the National Research Ethics Committee for the East of England (approval 00/237), and written informed consent was collected prior to any testing. The EEG recordings were always preceded by loudness-scaling measures so as to ensure that stimuli did not exceed a comfortable loudness. These measures were obtained using a 10-point chart labelled from 1 (just noticeable) to 10 (too loud) and where MCL (“most comfortable” loudness) corresponded to point 6, as described by Carlyon et al. [8]. Note that this loudness level may be softer than the maximum levels used in clinical settings.

**Table 1:** Participant details. Note that AB28, who was pre-lingually deaf, did not show any significant response at any level/modulation frequency and was not included in any of the data presented here

**ID**	**Experiment**	**Ear**	**Age**	**Implant**
**C26**	1 and 2	Right	58	Cochlear
**C27**	1	Right	71	Cochlear
**C28**	1	Right	31	Cochlear
**C30**	1 and 2	Right	73	Cochlear
**C34**	1 and 2	Left	69	Cochlear
**AB1**	1 and 2	Left	76	Advanced Bionics
**AB2**	2	Left	62	Advanced Bionics
**AB23**	2	Right	62	Advanced Bionics
**AB25**	2	Right	68	Advanced Bionics
**AB26**	2	Left	60	Advanced Bionics
AB28*	2	Left	42	Advanced Bionics
**AB31**	1	Right	56	Advanced Bionics

For experiments with the Cochlear device, stimuli were generated using the Nucleus Implant Communicator (NIC) software and research hardware provided by the manufacturer. One-second epochs of each stimulus were played continuously and without interruption; a trigger was produced at the start of each epoch, allowing the epochs to be averaged. Presentation for each stimulus continued until either 5 min (300 epochs) had elapsed or until at least 3 min had passed and with the amplitude at F0 being significantly above the noise floor and varying by less than 3 dB over the previous minute (cf. EEG analysis described at the end of this section).

For experiments with the Advanced Bionics (AB) device, stimulus generation was the same as for the Cochlear device except as follows. Stimuli were presented using routines from the Bionic Ear Data Collection System software (BEDCS 1.18.337, Advanced Bionics) and research hardware provided by the manufacturer. This version of the research software only allows one trigger to be sent at the very beginning of the stimulation rather than every second as was possible with the Cochlear device. Stimuli were therefore presented in 17-s blocks within which each 1-s epoch was repeated continuously and without interruption, and with a gap of about 1 s between each block. In order to cut the EEG signal into 1-s epochs without access to triggers, we detected and counted pulses in the raw data. The first 1-s block for each epoch was discarded so as to remove the influence of any onset effects.

The EEG system, montage, and recording methods were essentially the same as described by Carlyon et al. [8] with the exception that real-time analysis was made possible. The EEG system consisted of an 8-channel 24-bit hyper-rate system produced by the BioSemi company to our specifications and with a sampling rate of 262,144 Hz. This very high sampling rate is important for some measures of the electrically evoked steady-state response (EASSR) that were additionally obtained in experiment 1 (see below) but is not essential for the ALFIES method. The 8 electrodes were located as follows: left mastoid, right mastoid, P9 (temporal, left), P10 (temporal, right), Iz (back), Cz (top, vertex), Fpz (pre-frontal, midline), Fz (frontal, midline).

The EEG analysis pipeline was identical to that described by Carlyon et al. [8]: for all recording channels, 1-s epochs were cut based on the median inter-trigger interval (to correct for the different clocks between the Biosemi system and the implant), averaged together (ignoring the first epoch in each presentation block), referenced to electrode Cz and passed through a fast Fourier transform (FFT) analysis with 1-Hz resolution (1-s analysis window). Unless specifically mentioned, we averaged the two mastoid channels and Iz together, and defined this average as our region of interest. The power in the FFT at F0, F1, and F2 Hz was compared with that of the adjacent 6 bins (3 bins (3 Hz) each side) so as to calculate the SNR [19]. An F ratio greater than 10.92 (*p* < 0.01, approx. 10 dB SNR, Table 1 in Dobie and Wilson) was deemed significant. Compared to Carlyon et al. [8], the analysis code was modified to allow for real-time plotting of the results, but without any modification to the analysis pipeline itself. To do such real-time analysis with minimum modifications to our “offline” analysis code, we set the Biosemi software to save EEG data into 100-MB data files (~ 15 s). Thus, every 15 s and once a new data file was finished saving, we could start analysing it with our “offline” analysis code. We computed the power and SNR in the FFT at F0, F1, and F2 Hz every 8 epochs with the Cochlear setup, and every block (16 epochs) for the AB setup. As mentioned above, this allowed us to stop stimulation in a given condition when at least 3 min had passed, the amplitude at F0 was significantly above the noise floor, and its amplitude had varied by less than 3 dB over the previous minute. Overall, we shortened the recording time in 26% of the conditions (15% to 3 min, and 11% to 4 min).

### Experiment 1: Effect of Inter-Pulse Interval

The main stimuli used in experiment 1 consisted of two interleaved 480-pps pulse trains amplitude modulated at F1 = 80 Hz and F2 = 120 Hz, respectively. The NDR was measured as a function of the IPI between adjacent pulses in the 80- and 120-Hz modulated pulse trains (Fig. 1B). IPIs throughout this article are defined as the shortest duration during the pulse train from the end of one pulse from the F1 pulse train to the start of the immediately following F2 pulse (Fig. 1B). In addition, for participants implanted with the Cochlear device only, we measured the NDR to 2160-pps interleaved pulse trains, modulated at 80 and 120 Hz, as used by Carlyon et al. [8]. We also measured the EASSR for most Cochlear participants, as described below.

#### Cochlear Device

The main stimuli consisted of interleaved 480-pps pulse trains in which the IPI was 8, 25, 50, 100, 173, 400, 600, or 984 µs, and where each pulse had a duration of 25 µs per phase separated by an 8-µs inter-phase gap (IPG). The shortest IPI tested (8 µs) was the minimum possible for this device. At the longest value (984 µs), the pulses were isochronous. We first obtained threshold (T) and MCL for an unmodulated pulse train having a rate of 960 pps; this is equivalent to two 480-pps pulse trains interleaved with IPI = 984 µs. We then independently modulated the two constituent 480-pps pulse trains at 80- and 120-Hz respectively, with the minima of the modulation pattern fixed at the previously obtained T level and the maxima adjusted until the participant reported that the stimulus loudness was at MCL. This was done separately for IPI = 8, 400, and 984 µs. The lowest MCL value obtained from each of these conditions was then used for all IPIs tested. For the stimulus consisting of two 2160-pps pulse trains, the IPI was 173 µs, so that the composite stimulus consisted of a modulated 4320-pps isochronous pulse train. Analogous to the 480-pps conditions, the minimum of the modulated waveform was at the T level for a 4320-pps pulse train and the maximum value was adjusted so that the stimulus loudness was judged to be at MCL.

For all Cochlear participants except C28, we additionally measured the EASSR to 480-pps pulse trains using the same method described by Carlyon et al. [8], which was in turn based on the one developed by Gransier et al. [20]. EASSRs were obtained for a carrier rate of 480 pps and for modulation rates of 37, 40, and 43 Hz. Similar to the ALFIES measurements, we first obtained T and MCL for an unmodulated 480-pps pulse train. We then modulated the 480-pps pulse train with the minima of the modulation pattern fixed at the previously obtained T level and the maxima adjusted until the participant reported that the stimulus loudness was at MCL. The analysis method [20] involved identifying and blanking the stimulus pulses in the recording and linearly interpolating across the blanked values. In all the analyses presented here, the blanked periods extended from 0.2 ms before to 0.8 ms after each pulse.

#### Advanced Bionics Device

The procedure and stimuli used for participants implanted with an Advanced Bionics CI were similar to that for the Cochlear listeners, except as follows. Each pulse had a phase duration of 43 µs and a zero IPG. The IPIs were 0, 22, 43, 86, 172, 345, 582, and 948 us. The three IPIs tested to select the levels to be used for all stimuli had IPIs of 0, 345, and 948 µs. These differences arose from technical limitations of the two devices. No measures were obtained from Advanced Bionics listeners with interleaved 2160-pps pulse trains due to limitations in the participant testing time available. EASSRs were also not obtained from the AB participants.

### Experiment 2: Amplitude Growth Functions

#### Cochlear Device

The stimuli consisted of two interleaved 480-pps pulse trains sinusoidally amplitude modulated at rates of F1 and F2 Hz and with an IPI of 8 µs, similar to the 8-µs-IPI condition of experiment 1. This short IPI was chosen so as to maximise the expected amplitude of the NDR. The phase duration and inter-phase gap were 25 and 8 µs respectively, as in experiment 1. The threshold (T) and MCL for an unmodulated version of this stimulus were obtained via loudness scaling, and the AM applied to the pulse trains used for EEG measures had minima at the T level for the unmodulated stimulus and maxima at a higher level such that the modulated stimulus (F1 = 80 Hz, F2 = 120 Hz) was at MCL. The NDR was then measured for F0s of 37, 40, and 43 Hz and by setting F1 and F2 to 2 and 3 times these values respectively, such that the group delay could be calculated from the slope of the phase-vs-frequency plot for the component at F0. As well as confirming that the measured NDR had a group delay consistent with a thalamic/cortical source [8], this allowed us to select, for the main part of the experiment, an F0 at which there was a substantial NDR at MCL. This was valuable because the amplitude of both the NDR [8] and the EASSR [20–22], which share a similar latency and amplitude distribution across scalp electrodes, can vary idiosyncratically across participants with modulation rate and because we wished to measure the NDR across as wide a range of levels as possible without the measures being limited by recording noise. The values of F0 chosen for each participant for the main (level-growth) part of the experiment are shown in Table 2(d), along with other participant-specific details. We measured the NDR at a range of levels, relative to the MCL obtained in the loudness scaling stage, expressed in clinical current units (CUs), where 1 CU is approximately 0.16 dB. For participants C26 and C34, these relative levels were +3, 0, −3, −6, −9, −12, and −15 CUs (approximately +0.5, 0, −0.5, −0.9, −1.4, −1.9, and −2.4 dB); for listener C30, we additionally included levels of −21 and + 6 CUs (approximately −3.3 and + 0.9 dB) re MCL. The levels were tested in a different quasi-random order for each participant.

**Table 2:** Participant-specific details of the stimulus parameters used in each experiment. Parameters common to all participants are described in the main text. Thresholds (T) and MCLs are expressed in dB re 1µA, rounded to the nearest dB. Delays in part (d) are in µs. Where participants took part in both experiments the same test electrode was used in each, with the exception of AB1 who was tested on electrode 3 in experiment 1 and on electrode 4 in experiment 2

**(a) Experiment 1, IPI conditions**						
ID	F0 (Hz)	Electrode	T	MCL		
AB1	40	3	44	51		
AB31	40	3	42	50		
C26	40	16	44	51		
C27	40	20	50	56		
C30	40	20	45	50		
C34	40	20	47	50		
C28	40	16	52	58		
**(b) Experiment 1, 2160 pps**						
ID	F0 (Hz)	Electrode	T	MCL		
C26	40	16	40	48		
C27	40	20	48	55		
C28	40	16	38	48		
C30	40	20	44	50		
C34	40	20	36	56		
**(c) Experiment 1, EASSR**						
ID	Electrode	T	MCL			
C26	16	42	45			
C27	20	44	46			
C30	20	42	44			
C34	20	43	45			
**(d) Experiment 2, level growth**						
ID	F0 (Hz)	Carrier rate (pps)	Electrode	IPI delay	T	MCL
AB1	37	1998	4	143	37	48
AB2	40	2160	4	124	36	47
AB23	43	2322	4	108	40	47
AB25	43	2322	4	108	38	48
AB26	40	2160	4	124	36	45
C26	40	480	16	8	44	51
C30	43	480	20	8	44	50
C34	37	480	20	8	46	50

#### Advanced Bionics Device

The stimuli used with patients implanted with the Advanced Bionics device had pulse rates similar to those used by Carlyon et al. [8]. This was done because for these participants experiment 2 was performed before experiment 1 and because we initially wished to confirm that an NDR could be obtained using the Advanced Bionics device; we therefore considered it prudent to use stimuli as similar as possible to those for which a reliable NDR had been previously reported [8]. The phase duration and inter-phase gap were 53 and 8 µs respectively. We first measured the NDR for interleaved pulse trains modulated at F0 = 37, 40, and 43 Hz and with pulse rates of 1988, 2160, and 2322 pps respectively; the maximum and minimum levels in the modulated stimulus were the same for all modulation rates and were determined using the same loudness scaling technique as for the Cochlear participants and so as to have a loudness equal to MCL. As in Carlyon et al. [8], the IPI was such that each combined pulse train was isochronous. We then selected an F0 (and corresponding pulse rate) that produced a sizeable NDR for the participant under test, and then measured the NDR at +0.5, 0, −0.5, −1, −1.5, −2, and −2.5 dB relative to the MCL. These F0s are shown in Table 2(d), along with other details specific to each participant.

## Results

### Experiment 1

**Fig. 2:**
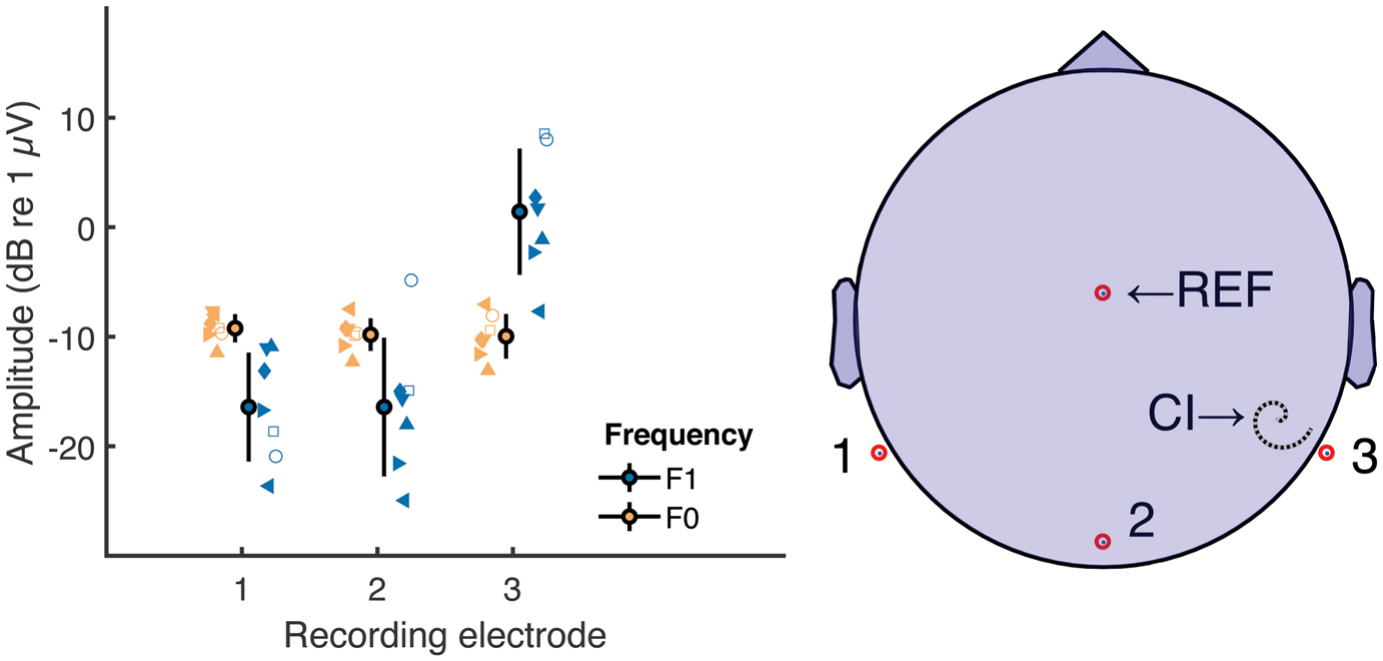
The left-hand plot shows the amplitude of the NDR (yellow) and F1-Hz component (blue) in experiment 1 and for three recording electrodes, as illustrated in the right-hand plot. For simplicity in this figure, we flipped the left/right electrodes for the participants that had their implant on the left side. In the left-hand plot, unconnected markers correspond to individual data (same marker type across all figures for each individual participant). Error bars show mean and standard deviation across all participants

The amplitudes of the FFT components at F0 and F1 Hz obtained with the shortest IPI used for each device are shown in Fig. 2 for three recording electrodes. The large symbols with error bars show the mean ± one standard deviation across participants, and small symbols show the data for individual participants. For this analysis “electrode 2” was Iz and electrodes 1 and 3 were contralateral and ipsilateral to the CI and at either P9 or P10, dependent on the side of implantation. The component at F0 Hz (yellow symbols)—corresponding to the NDR—is broadly independent of recording site, whereas the component at F1 (blue) is much greater for a recording electrode adjacent to, compared to distant from, the CI. This is consistent with the results described by Carlyon et al. [8] and with the conclusion that F0 reflects a neural component (the NDR), whereas F1 is dominated by electrical artefact. It extends that finding to show that a neural response at F0 can be recorded even with a very short IPI, namely 0 and 8 µs for the AB and Cochlear devices, respectively. The NDR amplitude was more than 10 dB above the noise floor for all participants.

**Fig. 3:**
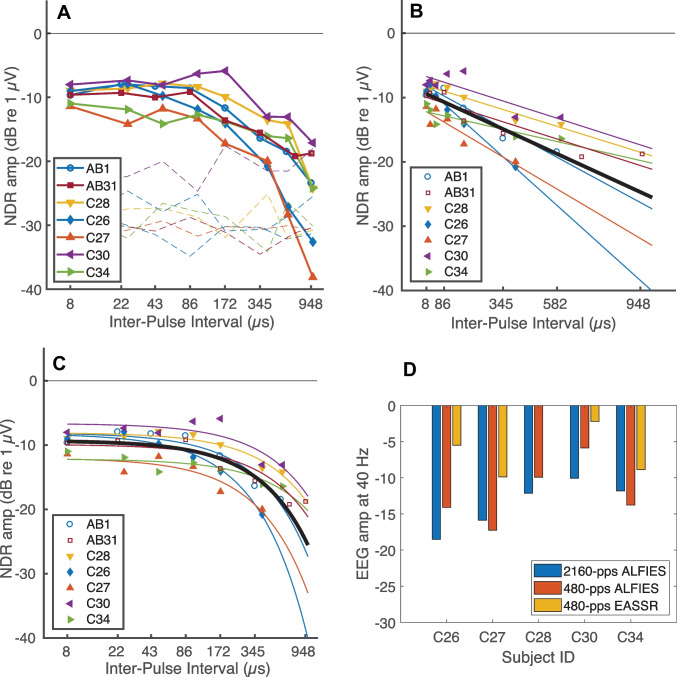
**A **Amplitude of the NDR as a function of IPI (solid line) with corresponding noise levels (dashed line). Data from AB and Cochlear participants are shown by open and filled markers respectively (same marker type across all figures for each individual participant). Parts **B** and **C **show log-linear fits to the data (ignoring data points not significantly above the noise floor) with IPI plotted on linear and logarithmic co-ordinates respectively. **D** The blue and red bars show the amplitude of the NDR obtained with the ALFIES method and an IPI of 173 µs and with the rate of each interleaved pulse train equal to 2160 and 480 pps respectively. Yellow bars show the EASSR amplitude for all participants except C28, from whom an EASSR was not obtained

The symbols connected by lines in Fig. 3A show the NDR amplitude as a function of IPI for the 480-pps pulse trains, averaged across electrodes at the two mastoids and Iz. For all participants, the NDR amplitude is roughly constant up to some value, above which it decreases. For most participants, it is below the noise floor at the longest IPI tested. There is no obvious marked difference between results obtained with the Cochlear and AB devices, shown by the filled and open symbols respectively. To further characterise the effect of IPI, we fitted the results for each participant using a linear slope relating the NDR amplitude in dB to IPI on a linear scale, ignoring nonsignificant data points. Because of the conversion between linear and logarithmic scale on the *x* axis, we set the lowest IPI to be 8 µs for all participants, including the AB ones for whom the actual IPI was 0 µs. The results (panels B and C in Fig. 3, log and linear scale on the *x* axis, respectively) show a 6-dB decrease in amplitude (compared to the 8-µs IPI) at an average of 457-µs IPI (ranging from 197 to 755 µs across participants).

The blue and red bars in Fig. 3D show the NDR amplitudes for the Cochlear participants with IPI = 173 µs at pulse rates of 480 pps (as in the main part of experiment 1) and at a rate of 2160 pps, as in Carlyon et al. [8]. It can be seen that there is no consistent difference in the NDR amplitudes obtained at the two pulse rates, and a paired-sample *t*-test revealed that the difference was not statistically significant (*t*(4) = 1.1, *p* = 0.33). The yellow bars show the amplitudes of the EASSRs obtained at 480 pps, which are consistently larger than that of the NDRs for the same four participants (i.e. all except C28, from whom no EASSR was obtained). This is consistent with Carlyon et al.’s [8] finding that EASSRs obtained at 500 pps were larger than NDRs obtained with ALFIES at a carrier rate 2160 pps, and shows that this is also true when ALFIES and EASSRs are obtained using similar pulse rates. Note, however, that at higher pulse rates it is not possible to measure EASSRs due to insufficient samples remaining after the artefact has been blanked.

### Experiment 2

**Fig. 4:**
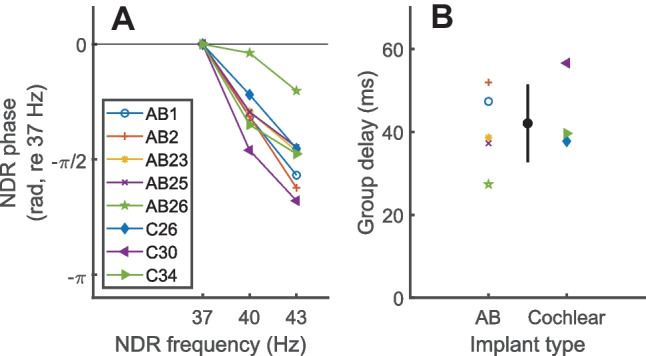
**A **NDR phase as a function of frequency from experiment 2, with data from AB and Cochlear participants shown by open and filled markers respectively. All points are referenced to the phase value at 37 Hz, for ease of visualisation (this does not impact the derivation of the group delay). **B** Coloured symbols show the group delay for AB (left) and Cochlear (right) participants. The black symbol with error bars shows the mean and standard deviation of the group delay calculated across all participants

Phase-vs-frequency plots for the F0 components are shown in Fig. 4A, with data from the Cochlear and AB participants shown by the filled and open symbols respectively. Participant AB28, who was long-term deaf, failed to show an NDR even at MCL and was excluded from the plots and analyses. Across the other participants, the mean group delay was 42 ms, with a standard deviation of 9.3 ms (Fig. 4B). This is consistent with the results of Carlyon et al. [8] and with the conclusion that F0 represents a neural response originating from the thalamus or auditory cortex [23–25]. Both the F1 and F2 components (not shown) produced near-zero group delays, consistent with them being dominated by an electrical artefact. A possible exception comes from the data of AB26, whose group delay of 27 ms was the shortest of all those tested and is consistent with a combination of artefact and more-central sources. Analysis of the data from individual recording electrodes showed that the NDR at the ipsilateral electrode was 5.9 dB larger than at the contralateral electrode—this difference is greater than in any other participant but much smaller than their average 23.3-dB difference in F1 amplitude. Overall, however, the results extend the demonstration of a sustained phase-locked thalamic/cortical response to high-rate pulse trains to AB participants and to stimuli with a short IPI of 0 µs (AB participants) or 8 s (Cochlear participants). As in experiment 1, but not shown here, the NDR amplitude was similar (averaged difference of 1.5 dB) for the different recording electrodes shown in Fig. 2 whereas the amplitudes at F1 and F2 were on average 20 dB larger ipsilateral than contralateral to the CI.

**Fig. 5:**
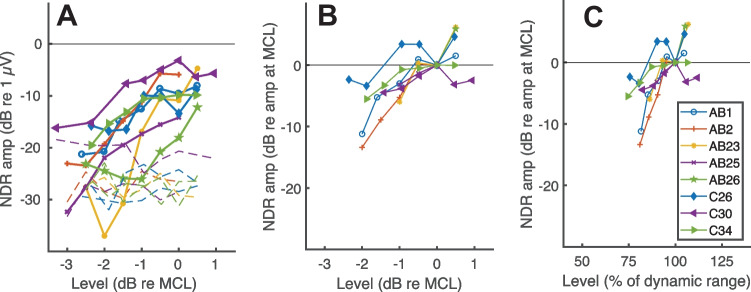
The solid lines with symbols in panel **A** show amplitude growth functions for the NDR plotted as stimulus amplitude in dB re MCL vs response amplitude in dB re 1 µV. The dashed lines without symbols show the noise floor for each participant and stimulus level. (Of the two blue dashed lines, the one with the higher noise floor at MCL and adjacent levels is for participant C26, and the other one is for AB1). Panel **B** shows these data normalised on both axes to the MCL for each listener, and with only data points above the noise floor plotted. Panel **C** shows the normalised response as a function of the stimulus level plotted as a percentage of the perceptual dynamic range in dB; again, only points above the noise floor are shown. Consistent marker types are used across all figures for individual participants

The solid lines with symbols in Fig. 5A and B show the NDR amplitude growth as a function of stimulus level in dB relative to MCL. In Fig. 5A (NDR amplitudes in dB re 1 µV), all data points are shown, and the noise levels are illustrated using dashed lines without symbols, while only data points that fell significantly above the noise floor are plotted in Fig. 5B (NDR amplitudes in dB relative to the amplitude at MCL). It can be seen that the growth functions are quite steep, and that, compared to MCL, the NDR at MCL-2 dB is reduced by at least 7 dB or is in the noise floor for all participants except C26. Although there are too few participants for each device to warrant a formal comparison, there is no obvious marked difference between Cochlear and AB listeners, plotted as filled and open symbols respectively, and despite the different pulse rates used for the two groups. Figure 5C shows the same data plotted as a function of each listener’s dynamic range (DR) in percent, calculated using T and MCL values in dB. For most participants, relative to MCL, the NDR for a stimulus presented at 75% of the DR is either below the noise floor (and hence not plotted) or reduced by at least 12 dB. One exception is listener C34 whose amplitude growth function shows a plateau for levels greater than −1 dB re MCL, corresponding to 75% of the DR. Even that participant, however, shows a 7-dB reduction for levels 2 dB lower than MCL, corresponding to 50% of their DR. In addition, listener C26 shows a nonmonotonic function and C30 shows a small reduction in the NDR at levels above MCL.

## Discussion

The results presented here are consistent with those described by Carlyon et al. [8] in showing that the ALFIES method can measure the sustained neural response to AM electrical pulse trains. We replicate the finding that this neural distortion response (NDR) has a group delay of about 40 ms, consistent with a thalamic/cortical source, and that it can be recorded even from electrodes close to the CI. We also extend those previous findings to measurements obtained with the Advanced Bionics device, measure the effect of inter-pulse interval on the NDR, and reveal the existence of generally steep level-growth functions.

### The Time Course and Neural Locus of NDR Generation

We previously argued that the generation of an NDR from interleaved pulse trains requires at least two processes [8]. The first process must involve some temporal interdependence in the neural responses to the interleaved F1 and F2 pulse trains. This requirement arises from the mathematical fact that the FFT is additive, and so the FFT to interleaved F1 and F2 pulse trains is equal to the sum of the FFTs to the (separate) F1 and F2 pulse trains. If each pulse train is subject to an *instantaneous* nonlinearity, then neither of these two individual FFTs will contain a component at any frequency that depends on both F1 and F2. However, neural systems introduce a number of temporal dependencies such that the response to each stimulus pulse is affected by previous pulses. Temporal dependencies in neural responses to electrical stimulation can take many forms, ranging from phenomena such as facilitation that arise from charge interactions at the level of the auditory nerve (AN), through to effects such as refractoriness and adaptation that occur both at the AN and throughout the brain [12]. As a result, the biological response at any one time is influenced by multiple previous pulses, corresponding to both the F1 and F2 pulse trains, and subsequent nonlinearities may then produce distortion components, such as F0 Hz, that depend on both F1 and F2. Note that the temporal dependency must occur *before* the nonlinearity: interleaved pulse trains subject to an instantaneous nonlinearity will not produce a component at any frequency that depends on both F1 and F2, and, for example subsequently smoothing the resulting waveform will not introduce such a component.

Because the time course of the different temporal dependencies such as facilitation, refractoriness, and adaptation differ from each other, and also vary throughout the auditory system, analysis of the effects of IPI on the NDR can constrain explanations of the underlying biological processes. The results of experiment 1 indicate the involvement of a process having a time constant of the order of about 400 µs. This value is consistent with the time course of facilitation that has been observed in animal experiments, as summarised by Boulet et al. [12]. Facilitation is the process by which an electrical pulse brings a neuron’s membrane close to generating an action potential, allowing a subsequent electrical pulse to trigger an action potential when it would not have done so in isolation [26]. It depends on the relative polarity of these adjacent pulses, and may be responsible for psychophysical phenomena that have been observed in human listeners and that also depend strongly on the relative polarity of adjacent pulses. For example, Karg et al. [10] measured the effect of a sub-threshold biphasic “pre-pulse” on the detection threshold of a subsequent biphasic “probe” pulse as a function of the inter-pulse gap. They reported significant threshold reductions that were greatest when the two pulses had opposite leading polarities, such that the last phase of the pre-pulse and the first phase of the probe had the same polarity. The condition with the largest reduction, which remained significant at a gap of 640 µs, occurred when these two phases had anodic polarity. Guérit et al. [14] used pairs of pseudomonophasic pulses, each consisting of a long low-amplitude phase and a short high-amplitude phase, and with one pulse time-reversed with respect to the other so that the two short-amplitude phases were adjacent. They found that, when the pulses in each pair had the same polarity, increasing the gap between the high-amplitude phases of the two pulses increased MCLs up to 4 dB, for gaps ranging from 0 to 173 µs, the longest gap studied. In contrast, in a study using similar stimuli but with pulses of opposite polarity, MCLs *decreased* with increasing gap up to 200 µs [13]. The dependence of these psychophysical phenomena on the relative polarities of two adjacent pulses is evidence that they reflect processes operating at the level of the AN, i.e. before the electrical charge has been converted to action potentials. However we should note that another short-term temporal dependency could arise in neurons in the cochlear nucleus that act as co-incidence detectors, firing only when there is near-synchronous firing of multiple AN fibres that form their input. This phenomenon has been observed both in octopus cells of the posteroventral cochlear nucleus [17, 18] and in bushy cells of the output tract of the anteroventral cochlear nucleus (i.e. the trapezoid body [15, 16]). In both cases, the co-incidence detection has a time constant of below 1 ms. In terms of the paradigm used in our experiment 1, the action potentials from AN fibres that fire to an F1 pulse could be combined with action potentials from other fibres that fire to the subsequent F2 pulse. The resulting firing pattern of the octopus or bushy cell would then depend on both F1 and F2, and hence any subsequent nonlinearity could then in principle produce an NDR at F0 Hz.

To summarise, we do not know exactly which processes introduce temporal dependencies necessary for the generation of an NDR but it is clear that the time-course of the results presented in Fig. 3 is consistent with processes known to operate at the level of the AN and/or cochlear nucleus. Once the effects of the interleaving have been undone, so that the responses to the F1 and F2 pulses are not independent, the subsequent nonlinearity necessary for NDR generation could then occur at any or all stages of the auditory system. One well-known neural nonlinearity occurs at the AN membrane: electrical stimuli (such as sinusoids or biphasic pulses) containing equal-sized positive and negative deflections can elicit action potentials, which are always highly asymmetric. It is also known that the function relating the firing rate of the AN to input current is not linear over a neuron’s entire dynamic range [27, 28]. However, other nonlinearities are clearly introduced at multiple stages from brainstem to cortex (e.g. modulation enhancement in the inferior colliculus [29]), and these stages will inherit any temporal dependencies occurring at the AN and/or cochlear nucleus. Hence, the results of experiment 1 primarily inform the stages of the auditory system at which the necessary temporal dependencies are likely to occur, rather than the locus of the nonlinearity responsible for the NDR. We can nevertheless conclude that more-central nonlinearities occur either after any temporal dependency that is sufficient to “undo” the effects of interleaving, or are of a form or size that does not elicit an NDR.

Finally, we consider an alternative explanation for the results of experiment 1. Carlyon et al. [8] performed measurements from cadaver heads, implanted with an Advanced Bionics CI, one electrode of which was stimulated with 80 + 120 Hz dyads (quantised approximations of 80- and 120-Hz sinusoids). When the 80- and 120-Hz stimuli were superimposed to produce the dyads, the EEG recording contained a component at F2-F1 Hz, which was substantially reduced but not eliminated by interleaving 126-µs segments of the F1 and F2 tones. It was suggested that some small amount of smoothing was introduced either by the electronics of the CI or by electro-chemical interactions between the stimulating electrode and the intra-cochlear saline. It is therefore possible that in the present experiments these physical effects generated a component at F0 at the electrode-cochlea interface, that was then propagated to the auditory thalamus/cortex which in turn generated the (approximately 40-Hz) neural activity detected by the EEG electrodes. According to this explanation, then, the response we recorded was neural but the smoothing and nonlinearity needed to generate it was purely physical. There are two reasons why this explanation is unlikely to be correct. First, the component recorded in cadavers disappeared when F1 and F2 were presented to adjacent electrodes rather than to the same electrode, whereas the NDR reported by Carlyon et al. [8] had the same size in these two conditions. Second, when a component at 40 Hz is physically introduced into the stimulus, as in EASSR measurements, the artefact at that frequency swamps the neural response unless steps (such as blanking or template subtraction) are taken to remove it [20, 30, 31]. No artefact removal was implemented or needed for the ALFIES technique in order to observe a neurally generated NDR near 40 Hz.

### Amplitude Growth Functions

We previously argued that the NDRs obtained using the ALFIES method could inform the setting of MCLs and/or thresholds in participants who are unable to provide reliable behavioural responses: the method is fast, does not require specialist equipment or complex analyses, can be obtained at the highest pulse rates used clinically and, being largely immune to electrical artefact, can be recorded even from electrodes close to the CI [8]. The present demonstration of generally steep amplitude growth functions, combined with the additional time savings afforded by real-time analysis, lends support to that assertion. A caveat is that two participants—C26 and C30—showed reductions in NDR with increases in stimulus amplitude over a small range. In both cases, this nonmonotonicity occurred at quite high stimulus levels, i.e. at MCL for C26 and at + 0.5 and + 0.9 dB re MCL for C30. The recorded NDRs at these levels were more than 10 dB above the noise floor for C26 and about 15 dB above the noise floor for C30, so we do not think that these findings arose from contamination of the responses by noise. Another reason why nonmonotonic growth functions can occur is when there are two different generators of different phases and whose relative influence varies as a function of level. Although we cannot completely rule out this possibility, and did not measure group delays as a function of level, there was no evidence that the NDR phase shifted at levels where these amplitude reductions occurred, compared to either higher or lower levels: For example, the phase of the NDR for C30 varied by less than 0.03 radians across the four levels between −0.5 and +0.9 dB re MCL. In the absence of any definitive explanation, we therefore simply note these exceptions to the more-common finding of monotonic functions obtained in the other 6 participants. It is also worth remarking that EASSR growth functions reported by Van Eckhoute et al. [32] for 7 participants did not show any nonmonotonicities near MCL, although their maximum level was limited to MCL and that the functions for seven of our eight participants (all except C26) were monotonic over this range.

## Summary

1. The use of the ALFIES method for measuring thalamic/cortical responses to electrical pulse trains was extended to users of the Advanced Bionics device. Additional time savings were achieved by implementing real-time analysis.
2. Manipulation of the interval between interleaved pulses revealed that the temporal dependency (“smoothing”) necessary for the generation of the NDR had a time constant of about 400–500 µs, consistent with facilitation at the level of the auditory-nerve membrane and/or co-incidence detection at the cochlear nucleus. The nonlinearity responsible for the NDR could have a locus at the auditory nerve and/or any neural site up to and including auditory cortex.
3. The NDR grows steeply and monotonically with increasing level, with the exception of two participants who showed a nonmonotonicity at stimulus levels at or above MCL.
4. EASSRs for 480-pps amplitude-modulated pulse trains are larger than NDRs obtained using the ALFIES method at the same pulse rate. This advantage should be balanced against the greater robustness of the ALFIES method to increases in pulse rate, recording-electrode position, and sampling rate of the EEG system, and to the ability of ALFIES to provide a measure of the temporal (experiment 1) and spatial [8] selectivity of the sustained thalamic/cortical response to the high-rate pulse trains typically used clinically.

## Data Availability

Data and analysis code is available upon request.

## References

[1] Middlebrooks JC, Snyder RL (2007). Auditory prosthesis with a penetrating nerve array. Jaro-Journal of the Association for Research in Otolaryngology.

[2] Dieter A, Keppeler D, Moser T (2020). Towards the optical cochlear implant: optogenetic approaches for hearing restoration. EMBO Mol Med.

[3] Brown CJ, Hughes ML, Luk B, Abbas PJ, Wolaver A, Gervais J (2000). The relationship between EAP and EABR thresholds and levels used to program the nucleus 24 speech processor: data from adults. Ear Hearing.

[4] Cafarelli Dees D (2005). Normative findings of electrically evoked compound action potential measurements using the neural response telemetry of the nucleus CI24M cochlear implant system. Audiology and Neurotology.

[5] McKay CM, Henshall KR, Hull AE (2005). The effect of rate of stimulation on perception of spectral shape by cochlear implantees. J Acoust Soc Am.

[6] Potts LG, Skinner MW, Gotter BD, Strube MJ, Brenner CA (2007). Relation between neural response telemetry thresholds, T- and C-levels, and loudness judgments in 12 adult nucleus 24 cochlear implant recipients. Ear Hearing.

[7] Macherey O, Stahl P, Intartaglia B, Meunier S, Roman S, Schon D (2021). Temporal integration of short-duration pulse trains in cochlear implant listeners: psychophysical and electrophysiological measurements. Hear Res.

[8] Carlyon RP, Guérit F, Deeks JM, Harland A, Gransier R, Wouters J, de Rijk SR, Bance ML (2021) Using interleaved stimulation to measure the size and selectivity of the sustained phase-locked neural response to cochlear-implant stimulation. J Assoc Res Otolaryngol. 10.1007/s10162-020-00783-y. 10.1007/s10162-020-00783-y PMC7943679 33492562

[9] de Balthasar C, Boëx C, Cosendai G, Valentini G, Sigrist A, Pelizzone M (2003) Channel interactions with high-rate biphasic electrical stimulation in cochlear implant subjects. Hear Res 182:77–87 10.1016/s0378-5955(03)00174-6 12948604

[10] Karg SA, Lackner C, Hemmert W (2013). Temporal interaction in electrical hearing elucidates auditory nerve dynamics in humans. Hear Res.

[11] Cosentino S, Deeks JM, Carlyon RP (2015). Procedural factors that affect measures of spatial selectivity in cochlear implant users. Trends in Hearing.

[12] Boulet J, White M, Bruce IC (2016). Temporal considerations for stimulating spiral ganglion neurons with cochlear implants. Jaro-Journal of the Association for Research in Otolaryngology.

[13] Guérit F, Marozeau J, Deeks JM, Epp B, Carlyon RP (2018). Effects of the relative timing of opposite-polarity pulses on loudness for cochlear implant listeners. J Acoust Soc Am.

[14] Guérit F, Marozeau J, Epp B, Carlyon RP (2020). Effect of the relative timing between same-polarity pulses on thresholds and loudness in cochlear implant users. Jaro-Journal of the Association for Research in Otolaryngology.

[15] Joris PX, Carney LH, Smith PH, Yin TCT (1994). Enhancement of neural synchronization in the anteroventral cochlear nucleus.1. Responses to tones at the characteristic frequency. J Neurophysiol.

[16] Joris PX, Smith PH, Yin TCT (1994). Enhancement of neural synchronization in the anteroventral cochlear nucleus.2. Responses in the tuning curve tail. J Neurophysiol.

[17] Golding NL, Robertson D, Oertel D (1995). Recordings from slices indicate that octopus cells of the cochlear nucleus detect coincident firing of auditory nerve fibers with temporal precision. J Neuroscience.

[18] Golding NL, Oertel D (2012). Synaptic integration in dendrites: exceptional need for speed. J Physiol.

[19] Dobie RA, Wilson MJ (1996). A comparison of t test, F test, and coherence methods of detecting steady-state auditory-evoked potentials, distortion-product otoacoustic emissions, or other sinusoids. J Acoust Soc Am.

[20] Gransier R, Carlyon RP, Wouters J (2020). Electrophysiological assessment of temporal envelope processing in cochlear implant users. Sci Rep.

[21] Gransier R, Deprez H, Hofmann M, Moonen M, van Wieringen A, Wouters J (2016). Auditory steady-state responses in cochlear implant users: Effect of modulation frequency and stimulation artifacts. Hear Res.

[22] Gransier R, Guérit F, Carlyon RP, Wouters J (2021). Frequency following responses and rate change complexes in cochlear implant users. Hear Res.

[23] Herdman AT, Lins O, Roon PV, Stapells D, Picton TW (2002). Intracerebral sources of human auditory steady-state responses. Brain Topogr.

[24] Farahani ED, Goossens T, Wouters J, van Wieringen A (2017). Spatiotemporal reconstruction of auditory steady-state responses to acoustic amplitude modulations: Potential sources beyond the auditory pathway. Neuroimage.

[25] Luke R, De Vos A, Wouters J (2017). Source analysis of auditory steady-state responses in acoustic and electric hearing. Neuroimage.

[26] Lapicque L (1907). Recherches quantitatives sur l’excitation électrique des nerfs traitée comme une polarisation. J Physiol Pathol Générale.

[27] van den Honert C, Stypulkowski PH (1987). Temporal response patterns of single auditory-nerve fibers elicited by periodic electrical stimuli. Hear Res.

[28] Miller CA, Abbas PJ, Robinson BK, Rubinstein JT, Matsuoka AJ (1999). Electrically evoked single-fiber action potentials from cat: responses to monopolar, monophasic stimulation. Hear Res.

[29] Krishna BS, Semple MN (2000). Auditory temporal processing: responses to sinusoidally amplitude-modulated tones in the inferior colliculus. J Neurophysiol.

[30] Hofmann M, Wouters J (2010). Electrically evoked auditory steady state responses in cochlear implant users. Jaro-Journal of the Association for Research in Otolaryngology.

[31] Hofmann M, Wouters J (2012). Improved electrically evoked auditory steady-state response thresholds in humans. Jaro-Journal of the Association for Research in Otolaryngology.

[32] Van Eeckhoutte M, Wouters J, Francart T (2018). Electrically-evoked auditory steady-state responses as neural correlates of loudness growth in cochlear implant users. Hear Res.

